# Dynamics of bacterial populations during bench‐scale bioremediation of oily seawater and desert soil bioaugmented with coastal microbial mats

**DOI:** 10.1111/1751-7915.12326

**Published:** 2016-01-11

**Authors:** Nidaa Ali, Narjes Dashti, Samar Salamah, Naser Sorkhoh, Husain Al‐Awadhi, Samir Radwan

**Affiliations:** ^1^ Microbiology ProgramDepartment of Biological SciencesFaculty of ScienceKuwait UniversityPO Box 5969Safat13060Kuwait

## Abstract

This study describes a bench‐scale attempt to bioremediate Kuwaiti, oily water and soil samples through bioaugmentation with coastal microbial mats rich in hydrocarbonoclastic bacterioflora. Seawater and desert soil samples were artificially polluted with 1% weathered oil, and bioaugmented with microbial mat suspensions. Oil removal and microbial community dynamics were monitored. In batch cultures, oil removal was more effective in soil than in seawater. Hydrocarbonoclastic bacteria associated with mat samples colonized soil more readily than seawater. The predominant oil degrading bacterium in seawater batches was the autochthonous seawater species *M* *arinobacter hydrocarbonoclasticus*. The main oil degraders in the inoculated soil samples, on the other hand, were a mixture of the autochthonous mat and desert soil bacteria; *X* *anthobacter tagetidis*, *P* *seudomonas geniculata*, *O* *livibacter ginsengisoli* and others. More bacterial diversity prevailed in seawater during continuous than batch bioremediation. Out of seven hydrocarbonoclastic bacterial species isolated from those cultures, only one, *M* *ycobacterium chlorophenolicum*, was of mat origin. This result too confirms that most of the autochthonous mat bacteria failed to colonize seawater. Also culture‐independent analysis of seawater from continuous cultures revealed high‐bacterial diversity. Many of the bacteria belonged to the *A* *lphaproteobacteria*, *F* *lavobacteria* and *G* *ammaproteobacteria*, and were hydrocarbonoclastic. Optimal biostimulation practices for continuous culture bioremediation of seawater via mat bioaugmentation were adding the highest possible oil concentration as one lot in the beginning of bioremediation, addition of vitamins, and slowing down the seawater flow rate.

## Introduction

Remediation of sites contaminated with xenobiotic compounds is achieved by physical and chemical methods, e.g. land filling and incineration (Kuiper *et al*., 2004). However, the physical removal of pollutants from all contaminated sites on earth is obviously very costly (Rosenberg, 1993). In addition, incineration is associated with air pollution, and land filling frequently leads to leachates in the form of gases and liquids which can pollute the ground water (Kuiper *et al*., 2004). The much more cost‐effective and more environmentally friendly technology of bioremediation implies the use of microbial activities in pollutant biodegradation (Atlas and Pramer, 1990). It comprises two major practices. ‘Bioaugmentation’ (inoculation or seeding), which implies the introduction of suitable oil‐degrading microorganisms into the contaminated site. The second practice is ‘biostimulation’, whose objective is to enhance the activities of indigenous (autochthonous) pollutant‐degrading microorganisms via environmental management, e.g. the addition of nutrients and other growth‐limiting factors, especially nitrogen and phosphorus (Atlas and Bartha, 1998; Radwan, 2009). Bioremediation commonly is recommended as an alternative technology to the use of chemicals and other toxic materials for removing hydrocarbon contaminants (Piskonen and Itävaara, 2004).

As already mentioned, bioaugmentation implies the inoculation of the contaminated sites with laboratory grown, hydrocarbon‐degrading microorganisms (Al‐Awadhi *et al*., 1996; Van Limbergen *et al*., 1998; Kuiper *et al*., 2004). This leads to the introduction of additional gene pools complementary to the already existing ones, with the purpose of enhancing degradation of contaminants (Domde *et al*., 2007). In a study on the effect of bioaugmentation with a consortium of bacteria on the remediation of hydrocarbon contaminated waste water, the water chemical oxygen demand, which reflects the organic substance content, dramatically decreased (Domde *et al*., 2007). Obviously, the proper consortia of microorganisms should be used in order to complete the degradation process (Kapley and Purohit, 2001; Moharikar *et al*., 2003; Domde *et al*., 2007). In literature reports, exogenous pure cultures as well as unidentified mixtures of microorganisms have been used for bioaugmentation (Atlas and Bartha, 1998). Based on their ability to degrade a wide range of organic compounds, species of *Pseudomonas* have been frequently selected (Atlas and Bartha, 1998). Evidently, the bioaugmented organisms should be adapted to physicochemical parameters of the contaminated site. Imported *Arthrobacter* strains, in contrast to locally isolated ones, failed to colonize local oil‐polluted soils due to their inability to compete with the already existing strains (Radwan *et al*., 1997). Although proper microorganisms may be inoculated, they may fail to remove the pollutant (El Fantroussi and Agathos, 2005). Reportedly, this could be due to the absence of a single bacterium that possesses the entire set of enzymes needed to biodegrade the pollutant. Another five reasons have been suggested (Goldstein *et al*., 1985): the contaminant concentration is too low to support bacterial growth, presence of inhibitors that suppress microbial growth and/or activity, reduction of bacterial numbers due to protozoan grazing, presence of better utilizable sources of carbon and inability of the microbial cells to spread and reach the pollutant.

Biostimulation, the second bioremediation, practice implies, among others, the addition of nutrients, usually nitrogen, phosphorous and trace elements (Korda *et al*., 1997). Enhancing effects of biostimulation on hydrocarbon biodegradation have been documented (Bossert and Bartha, 1984; Leahy and Colwell, 1990; Atlas, 1991; Margesin and Schinner, 1998, Namkoong *et al*., 2002, Jimenez *et al*., 2007; For review see Nikolopoulou and Kalogerakis, 2009). On the other hand, a few investigators found that the rate of hydrocarbon degradation was not affected following the addition of nutrients (Seklemova *et al*., 2001). It has been reported that the percentage of oil degraded was inversely proportional to the concentration of the contaminating oil (Rahman *et al*., 2002). Bioremediation in the field is unpredictable because of the lack of knowledge of the persisting microorganisms in the site (Head, 1998).

Ideally, biostimulation should be coupled with bioaugmentation (Odokuma and Dickson, 2003; Coppotelli *et al*., 2008; Nikolopoulou *et al*., 2013a,b). When the efficiency of bioaugmentation and biostimulation in Long Beach soils and Hong Kong soils was compared, it was found that biostimulation achieved more hydrocarbon removal (Bento *et al*., 2005). However, there is no feasible technology for enhancing nutrient availability in the open seas (Rosenberg, 2006). Evidently, autochthonous microorganisms are to be chosen for bioaugmentation (Hosakawa *et al*., 2009). In view of the fact that biostimulation enhances autochthonous microorganism (DiGregorio *et al*., 2015), the two practices (autochthounous bioaugmentation and biostimulation) could be regarded as two faces of one coin. Autochthonous microorganisms of a habitat are the natural inhabitants, contributing to biochemical activities therein. Their counterparts, the allochthonous microorganisms are foreign survivals which do not contribute significantly to activities in the habitat.

The following snap shots summarize the history of the ‘autochthonous bioaugmentation (ABA)’ concept, and contribute to highlighting the objectives of this study. About two and half decades back, one of our group (Radwan, 1991) warned from using imported microbial cocktails, instead of depending on indigenous microorganisms for combating the greatest man‐made oil spill in the history of mankind (the spill associated with the 1990–1991 occupation of Kuwait by the Iraqi forces). Experimental studies supported the validity of this concept (Vecchioli *et al*., 1990; Weber and Corseuil, 1994). However, it was Ueno and colleagues (2007) who coined the term ‘autochthonous bioaugmentation (ABA)’, which necessitates the use of natural microbial inhabitants of an environment for its bioremediation. With this background in mind, the major objective of this paper was to study, in bench‐scale experiments, the feasibility of using local microbial mats from Kuwaiti coasts, instead of laboratory‐grown microbial cocktails, as bioaugmentation materials for bioremediation of local oil‐contaminated seawater and desert soil samples. We selected microbial mats on the basis of our earlier report (Sorkhoh *et al*., 1992) that they were the primary colonizers of coastal oil sediments, and consequently the first sign of self‐cleaning of the dead coasts that had been heavily polluted during the Iraqi occupation of Kuwait. Reportedly, such coastal mats were rich in hydrocarbonoclastic bacteria, well adapted to the Kuwaiti conditions. There are still only a very few studies worldwide on the ABA strategy (Hosakawa *et al*., 2009), and almost none on the contaminated Kuwaiti habitats. These facts highlight the need for the current study.

## Results

### Oil removal in batch culture

**Figure 1:**
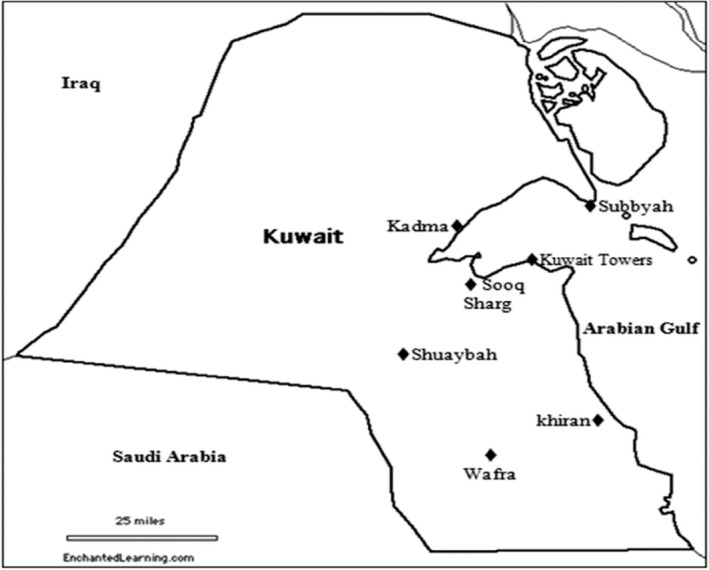
Kuwait map showing the sampling sites of coastal mats, seawater and desert soil samples.

The Kuwait map in Fig. 1 shows where the environmental samples have been taken.

**Figure 2:**
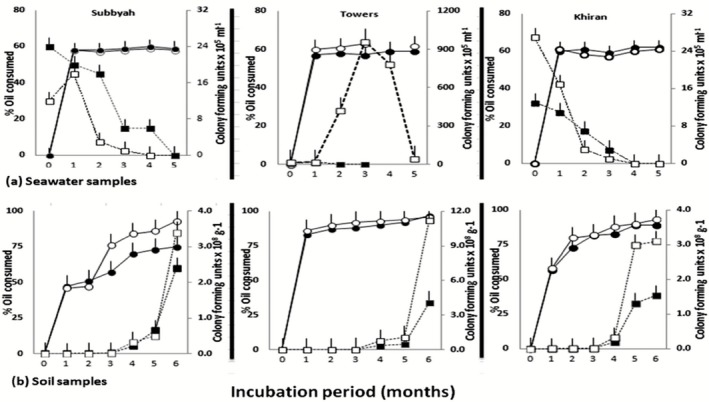
Oil consumption and numbers of cultivable hydrocarbonoclastic bacteria during bioremediation of seawater and desert soil samples in batch cultures using microbial mats for bioaugmentation. Solid lines, oil consumption; broken lines, bacterial numbers; closed symbols, sterile samples; open symbols, fresh samples.

The results in Fig. 2 show that about 60% of the oil in the seawater batches were consumed after the first month of incubation. The consumption values did not increase thereafter. There were also no marked differences between the consumption values obtained from the sterilized and unsterilized seawater samples. It will be shown soon that the typical seawater bacterium *Alcanivorax hydrocarbonoclasticus* was the active organism in both samples. It reached the sterilized seawater with the bioaugmented mat which was suspended in seawater.

In the desert soil batches, oil consumption increased from time zero, reaching maximum values between the third and sixth months. Higher oil removal values were commonly measured in soil batches than in seawater batches.

### Numbers of hydrocarbonoclastic bacteria in batch cultures

With the exception of the unsterilized seawater batches from the Kuwait Towers, whose bacterial numbers increased reaching a maximum in month 3, the numbers of bacteria in the seawater samples were highest after the first month, and decreased with prolonged incubation (Fig. 2). In several batches, the bacteria died after 4 months, as indicated by the stinky, anaerobic smell. In the soil batches, the numbers of the hydrocarbonoclastic bacteria kept increasing from time zero till the end of the 6‐month bioremediation period.

### 16S rRNA gene sequencing for hydrocarbonoclastic bacterial isolates from batch cultures

**Figure 3:**
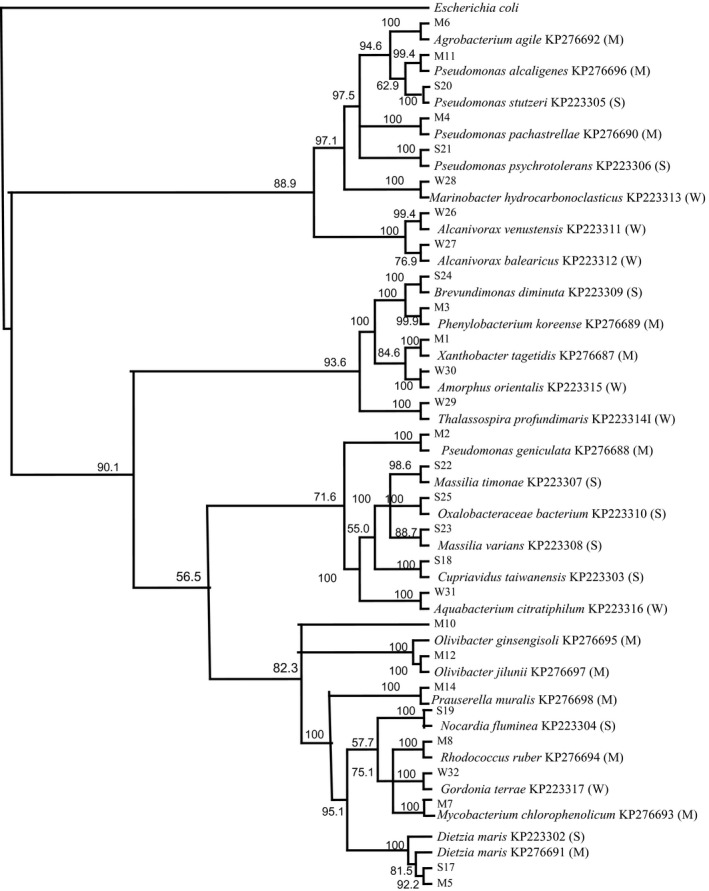
16S rRNA gene phylogeny of 28 hydrocarbonoclastic bacterial isolates from mat, soil and seawater. Values shown in each node of the tree are bootstrap values; 2000 bootstrap replicates were performed. M, microbial mat; S, desert soil; W, sea water

**Figure 4:**
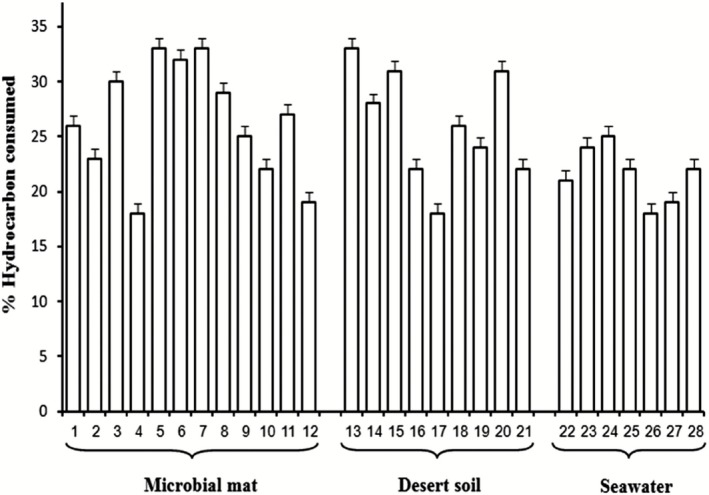
Oil consumption values by autochthonous bacterial isolates from three habitats. Values are means of three replicates. 1; *X* *anthobacter tagetidis*, 2*; Pseudomonas geniculata*, 3*; Phenylobacterium koreense*, 4*; P. pachastrellae*, 5*; D. maris*, 6*; Agrobacterium agile*, 7*; M. chlorophenolicum*, 8*; R. ruber*, 9*; Olivibacter ginsengisoli*, 10*; P. alcaligenes*, 11*; O. jilunii*, 12*; P. muralis*, 13*; Dietzia maris*, 14*; Cupriavidus taiwanensis*, 15*; Nocardia fluminea*, 16*; Pseudomonas stutzeri*, 17*; Pseudomonas psychrotolerans*, 18*; Massilia timonae*, 19*; Massilia varians*, 20*; Brevundimonas diminuta*, 21*; Oxalobacteraceae bacterium*, 22*; Alcanivorax venustensis*, 23*; Alcanivorax balearicus*, 24*; Marinobacter hydrocarbonoclasticus*, 25*; Thalassospira profundimaris*, 26*; Amorphus orientalis*, 27*; Aquabacterium citratiphilum*, 28*; Gordonia terrae.*

Table 1 presents the results of 16S ribosomal (r)RNA gene sequencing of the autochthonous, hydrocarbonoclastic bacteria isolated by the culture‐dependent method from coastal mat, desert soil and seawater samples. Most isolates showed 99–100% similarities in their sequences to their closest relatives in the GenBank database. Many of the autochthonous mat inhabitants were affiliated with the class of *Alphaproteobacteria*, with fewer members affiliated with the *Sphingobacteridae*, *Gammaproteobacteria* and *Actinobacteridae*. Autochthonous desert soil inhabitants and seawater inhabitants belonged predominantly to the *Actinobacteridae* and *Proteobacteria* respectively. In other words, autochthonous bacteria in the three habitats belong to diverse systematic taxa. The phylogenetic tree in Fig. 3 illustrates phylogenetic relationships among those hydrocarbonoclastic isolates. Figure 4 shows that the individual autochthonous bacterial isolates from the three habitats consumed in batch cultures within 14 d between about one fifth and one third of the crude oil.

**Table 1:** 16S rRNA gene sequencing of constituent hydrocarbonoclastic bacteria indigenous to microbial mats, seawater and desert soil

Isolates	Total bases	Subdivision	Nearest GenBank match	Similarity %	Bases compared	Accession numbers
Microbial mats						
M1	468	*Alphaproteobacteria*	*Xanthobacter tagetidis* strain TagT2C	99	475/478	KP276687
M2	514	*Gammaproteobacteria*	*i>Pseudomonas geniculata* strain KNUC2110	100	514/514	KP276688
M3	469	*i>Alphaproteobacteria*	*Phenylobacterium koreense* strain SBR9	99	480/485	KP276689
M4	514	*Gammaproteobacteria*	*Pseudomonas pachastrellae* strain mj02‐PW8‐OH9	99	517/518	KP276690
M5	484	*Actinobacteria*	*Dietzia maris* strain W13107	100	484/484	KP276691
M6	510	*Alphaproteobacteria*	*Agrobacterium agile*	100	510/510	KP276692
M7	490	*Actinobacteria*	*Mycobacterium chlorophenolicum* isolate 42C8	100	490/490	KP276693
M8	494	*Actinobacteria*	*Rhodococcus ruber* strain Z17‐3	100	494/494	KP276694
M10	458	*Sphingobacteriia*	*Olivibacter ginsengisoli* strain Gsoil 060	96	494/513	KP276695
M11	498	*Gammaproteobacteria*	*Pseudomonas alcaligenes* strain SM‐26	99	507/511	KP276696
M12	491	*Sphingobacteriia*	*Olivibacter jilunii* strain 14‐2A	99	500/504	KP276697
M14	487	*Actinobacteria*	*Prauserella muralis* strain 05‐Be‐005	99	489/490	KP276698
Desert soil						
S17	481	*Actinobacteria*	*Dietzia maris* strain DSM 43672	100	481/481	KP223302
S18	383	*Betaproteobacteria*	*Cupriavidus taiwanensis*	100	383/383	KP223303
S19	335	*Actinobacteria*	*Nocardia fluminea* strain S1	98	352/360	KP223304
S20	513	*Gammaproteobacteria*	*Pseudomonas stutzeri* strain ECP10	100	513/513	KP223305
S21	508	*Gammaproteobacteria*	*Pseudomonas psychrotolerans* strain ZAP069	99	511/512	KP223306
S22	503	*Betaproteobacteria*	*Massilia timonae* strain WK‐79s	99	508/510	KP223307
S23	496	*Betaproteobacteria*	*Massilia varians* strain E26 q‐63	99	506/510	KP223308
S24	457	*Alphaproteobacteria*	*Brevundimonas diminuta* strain 2P06AC	98	478/487	KP223309
S25	507	*Betaproteobacteria*	*Oxalobacteraceae bacterium* NR185	99	510/511	KP223310
Seawater						
W26	489	*Gammaproteobacteria*	*Alcanivorax venustensis* strain 2PR57‐5	99	498/503	KP223311
W27	499	*Gammaproteobacteria*	*Alcanivorax balearicus* strain G06‐163_VO	100	499/499	KP223312
W28	514	*Gammaproteobacteria*	*Marinobacter hydrocarbonoclasticus* strain SBU2	100	514/514	KP223313
W29	478	*Alphaproteobacteria*	*Thalassospira profundimaris* strain S8‐2	98	500/510	KP223314
W30	459	*Alphaproteobacteria*	*Amorphus orientalis* strain YIM D10	99	471/477	KP223315
W31	500	*Betaproteobacteria*	*Aquabacterium citratiphilum* strain B4	100	500/500	KP223316
W32	487	*Actinobacteria*	*Gordonia terrae* strain DSM 43249	99	489/490	KP223317

### Population dynamics of hydrocarbonoclastic bacteria during bioremediation in batch cultures

The three oil‐contaminated seaweater samples that had been bioaugmented with coastal mat were colonized by *Marinobacter hydrocarbonoclasticus*. In comparison, the three oil‐contaminated desert soil samples that had been bioaugmented with coastal mat exhibited much more diversity, as far as their hydrocarbonoclastic bacterial population was concerned (Table 2). Throughout the incubation period, this population consisted of a mixture of autochthonous mat and desert soil bacterial inhabitants. *Xanthobacter tagetidis*, *Pseudomonas geniculata* and albeit in much fewer numbers, *Olivibacter ginsengisoli* were found in all the soil samples throughout the incubation period. *Phenylobacterium koreense* formed considerable proportions of the total bacteria at time zero and after 1 month, but decreased in months 2 and 3. However, the population re‐increased in months 4 and 5. The proportions of *Agrobacterium agile*, *Mycobacterium chlorophenolicum* and *Pseudomonas alcaligenes* showed sharp fluctuation during the bioremediation process (Table 2). In addition, some of the soil samples contained one or more of the following hydrocarbonoclastic species in the proportions specified in Table 2 notes: *Pseudomonas pachastrellae*, *Dietzia maris*, *Rhodococcus ruber*, *Olivibacter jilunii* and *Prauserella muralis*. Evidently, the autochthonous mat bacterioflora colonized the soil batches more readily than the seawater batches. All the above species are hydrocarbonoclastic, as judged by their ability to grow on the mineral medium with oil vapor as a sole source of carbon and energy. As already mentioned, quantitative determinations revealed that those organisms consumed considerable proportions of the available oil (see Fig. 4).

**Table 2:** Dynamics of hydrocarbonoclastic bacterial populations in soil batches bioaugmented with microbial mats

	Kadma		Shuaybah		Wafra	
	Sterile	Fresh	Sterile	Fresh	Sterile	Fresh
Time zero						
*Xanthobacter tagetidis*	25*^(1)^	12*^(2)^	15*^(3)^	2*^(4)^	14*^(5)^	19*^(6)^
*Pseudomonas geniculata*	15	30	–	20	12	11
*Phenylobacterium koreense*	12	8	26	–	7	8
*Agrobacterium agile*	16	–	–	2	23	7
*Mycobacterium chlorophenolicum*	11	13	22	2	10	9
*Olivibacter ginsengisoli*	–	6	4	27	–	–
*Pseudomonas alcaligenes*	–	–	–	27	–	–
1 month						
*Xanthobacter tagetidis*	16*^(7)^	44	26	13*^(8)^	31	6*^(9)^
*Pseudomonas geniculata*	35	42	24	18	30	6
*Phenylobacterium koreense*	14	9	3	11	4	5
*Agrobacterium agile*	5	4	–	–	–	4
*Mycobacterium chlorophenolicum*	1	1	–	1	1	3
*Olivibacter ginsengisoli*	–	–	14	21	20	18
*Pseudomonas alcaligenes*	–	–	33	35	14	30
2 months						
*Xanthobacter tagetidis*	41*^(10)^	41	46*^(11)^	18	20	21
*Pseudomonas geniculata*	26	9	36	34	18	17
*Phenylobacterium koreense*	5	6	–	–	–	–
*Agrobacterium agile*	4	10	3	8	3	1
*Mycobacterium chlorophenolicum*	–	1	–	–	2	–
*Olivibacter ginsengisoli*	12	29	21	23	23	48
*Pseudomonas alcaligenes*	–	–	–	15	33	13
3 months						
*Xanthobacter tagetidis*	32*^(12)^	49*^(13)^	59*^(14)^	43	41*^(15)^	45*^(16)^
*Pseudomonas geniculata*	51	28	30	45	15	15
*Phenylobacterium koreense*	–	–	–	–	–	–
*Agrobacterium agile*	–	20	–	12	–	4
*Mycobacterium chlorophenolicum*	3	1	–	1	–	–
*Olivibacter ginsengisoli*	–	–	–	–	–	–
*Pseudomonas alcaligenes*	–	–	–	–	–	30
4 months						
*Xanthobacter tagetidis*	38	26*^(17)^	20	72	9*^(18)^	35
*Pseudomonas geniculata*	55	33	7	4	13	55
*Phenylobacterium koreense*	4	4	16	4	11	10
*Agrobacterium agile*	–	–	–	–	–	–
*Mycobacterium chlorophenolicum*	–	1	–	–	1	–
*Olivibacter ginsengisoli*	–	8	25	20	8	–
*Pseudomonas alcaligenes*	–	24	26	–	51	–
5 months						
*Xanthobacter tagetidis*	14*^(19)^	16	10	16	11	23
*Pseudomonas geniculata*	26	14	36	35	6	33
*Phenylobacterium koreense*	11	7	8	–	60	–
*Agrobacterium agile*	16	–	–	8	–	–
*Mycobacterium chlorophenolicum*	–	–	–	–	8	–
*Olivibacter ginsengisoli*	16	20	17	26	15	34
*Pseudomonas alcaligenes*	–	31	36	–	–	–

Values are % of total cfu, *^1)^ + *Pseudomonas pachastrellae* (13%) + *Dietzia maris* (4%), *Rhodococcus Ruber* (4%); *^2^^)^ + *P. pachastrellae* (11%), *D. maris* (6%), *R. ruber* (13%); *^3^^)^ + *P. pachastrellae* (10%), *D. maris* (11%), *R. ruber*(11%), *^4)^ + *P. pachastrellae*(2%), *R. ruber*(1%), *Olivibacter jilunii* (19%); *^5)^ + *P. pachastrellae* (13%), *D. maris* (14%), *R. ruber* (5%); *^6)^ + *P. pachastrellae* (14%), *D. maris* (13%), *R. ruber* (5%), *Prauserella muralis* (9%); *^7)^ + *P. pachastrellae* (2%), *D. maris* (2%), *R. ruber* (1%), *O. jilunii* (23%); *^8)^ *P. pachastrellae* (1%), *^9)^ *D. maris* (2%), *O. jilunii* (25%); *^10)^ + *P. pachastrellae* (3%); *^11)^ + *P. pachastrellae* (1%); *^12)^ + *O. jilunii* (13%); *^13)^ *P. pachastrellae* (1%); *^14)^ + *P. pachastrellae* (11%); *^15)^ + *D. maris* (4%), *O. jilunii* (40%), *^16)^ + *D. maris* (6%); *^17)^ + *P. pachastrellae* (4%); *^18)^ + *O. jilunii* (6%); *^19)^ *O. jilunii* (8%).

### Oil consumption and numbers of hydrocarbonoclastic bacteria in continuous cultures

In culture vessels of the six chemostat‐like units, the crude oil remained as separate phases for 2 to 3 d, after which it dispersed in the water, probably via extracellular biosurfactants. A small proportion remained as droplets adhering to the vessel walls and connection tubes. However, all the residual oil in the individual units was completely recovered and analysed, as described in the experimental part.

**Figure 5:**
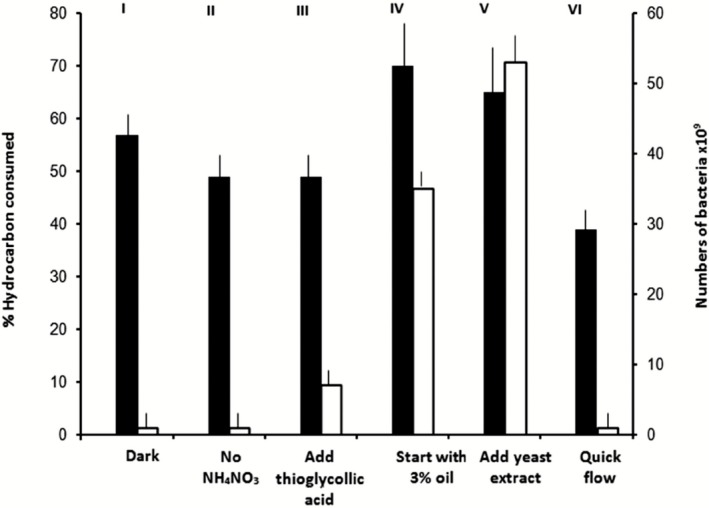
Oil consumption values (closed columns) and hydrocarbonoclastic bacterial numbers (open columns) in the culture vessels of six chemostat‐like units with various treatments.

The results in Fig. 5 show that starting the bioremediation process by adding the whole 3% oil as one lot at time zero resulted in the maximum oil removal (70%, unit IV). As described in the experimental part, the 3% oil had been added in all other chemostat‐like units as six 0.5% aliquots at 2‐week intervals. Addition of yeast extract as a vitamin source (unit V), was associated with 65% oil removal. The lowest oil consumption value of 39% was obtained when the seawater flow rate (unit VI) was five times quicker (30 ml h^−1^) than in all the other five chemostat‐like units. Neither the addition of the reducing substance thioglycollic acid (unit III), nor the deletion of NH_4_NO_3_ (unit II) were associated with any dramatic reduction of oil removal, which amounted to about 50%. Oil removal was also quite effective (57%) in the dark‐incubated culture vessel (unit I). All the seawater samples in the six chemostat‐like units at the end of bioremediation were rich in hydrocarbonoclastic bacteria. The numbers of the cfu ml^−1^ were in the magnitudes of 10^9^ and 10^10^, compared with only 10^5^ at time zero. The highest bacterial numbers were associated with the highest oil removal values (units IV and V).

### 16S rRNA gene sequencing for hydrocarbonoclastic bacteria from continuous cultures

Table 3 presents the results of 16S rRNA gene sequencing of hydrocarbonoclastic bacteria isolated by plating from the six chemostat‐like units after incubation for 12 weeks. The seven bacterial isolates exhibited sequence similarities of 99% and 100% to the closest GenBank relatives; they were affiliated with the *Actinobacteridae* and *Gammaproteobacteria*.

**Table 3:** 16S rRNA gene sequencing of hydrocarbonoclastic bacteria isolated from continuous cultures by the culture‐dependent method

Isolates	Total bases	Subdivision	Nearest GenBank match	Similarity %	Bases compared	Accession numbers
CC1	495	*Actinobacteria*	*Mycobacterium chlorophenolicum* isolate 42C8	99	497/498	KP276680
CC2	509	*Gammaproteobacteria*	*Vibrio parahaemolyticus* strain DAHMV3	100	509/509	KP276681
CC3	509	*Gammaproteobacteria*	*Vibrio diabolicus* strain KM30‐12‐3	99	512/514	KP276682
CC4	484	*Actinobacteria*	*Dietzia maris* strain NITD_PL2	100	484/484	KP276683
CC5	507	*Gammaproteobacteria*	*Alcanivorax dieselolei* strain NIOT‐Ba‐7	100	507/507	KP276684
CC6	494	*Actinobacteria*	*Gordonia bronchialis* strain A5‐8	99	500/503	KP276685
CC7	506	*Actinobacteria*	*Gordonia terrae* strain 5‐Sj‐4‐3‐2‐M	100	506/506	KP276686

### Composition of the bacterial populations in the six chemostat‐like units

Table 4 shows that out of the seven different hydrocarbonoclastic bacterial species identified, only three to five were found in the individual reaction vessels that had been subjected to the studied cultural variables. *Vibrio parahaemolyticus, V. diabolicus*, *Alcanivorax dieselolei* and *Mycobacterium chlorophenolicum* shared the predominance in the dark‐incubated unit I. The 2 *Vibrio* spp. and *M. chlorophenolicum* in addition to *Gordonia bronchialis* shared the predominance in the NH_4_NO_3_‐deprived seawater samples (unit II). *Vibrio parahaemolyticus* and *D. maris* contributed more than 50% of the total bacterial species in the thioglycollic‐acid‐amended vessel (unit III). In unit IV, which had received the whole 3% oil lot at time zero, *M. chlorophenolicum*, *V. parahaemolyticus*, *D. maris* and *G. terrae* shared the predominance. In the yeast extract‐amended seawater (unit V), *D. maris* formed > 60%, and *M. chlorophenolicum* was about 24% of the total. In unit VI with the quick water flow rate, *G. bronchialis*, *D. maris* and *G. terrae* shared the predominance.

**Table 4:** Composition of the hydrocarbonoclastic bacterial populations in oily seawater at the end of continuous culture bioremediation as analysed by the culture‐dependent method

Isolates	% of cfu of total hydrocarbonoclastic bacteria after bioremediation for 12 weeks					
	Dark incubated	No NH_4_NO_3_ added	+ Thioglycollate	3% Oil from time zero	+ Yeast extract	Quick flow rate
*Mycobacterium chlorophenolicum*	18.7	22.7	–	26.2	24.7	–
*Vibrio parahaemolyticus*	26.8	26	30.4	26.4	14.8	–
*Vibrio diabolicus*	24	29.1	6.5	6.4	–	4.4
*Dietzia maris*	8.1	–	26.8	20.1	60.4	27.4
*Alcanivorax dieselolei*	22.2	–	13.7	–	–	2.4
*Gordonia bronchialis*	–	22.2	7.2	–	–	38.7
*Gordonia terrae*	–	–	–	20.6	–	26.9

### Culture‐independent analysis of the total bacteria in the six chemostat‐like units

**Figure 6:**
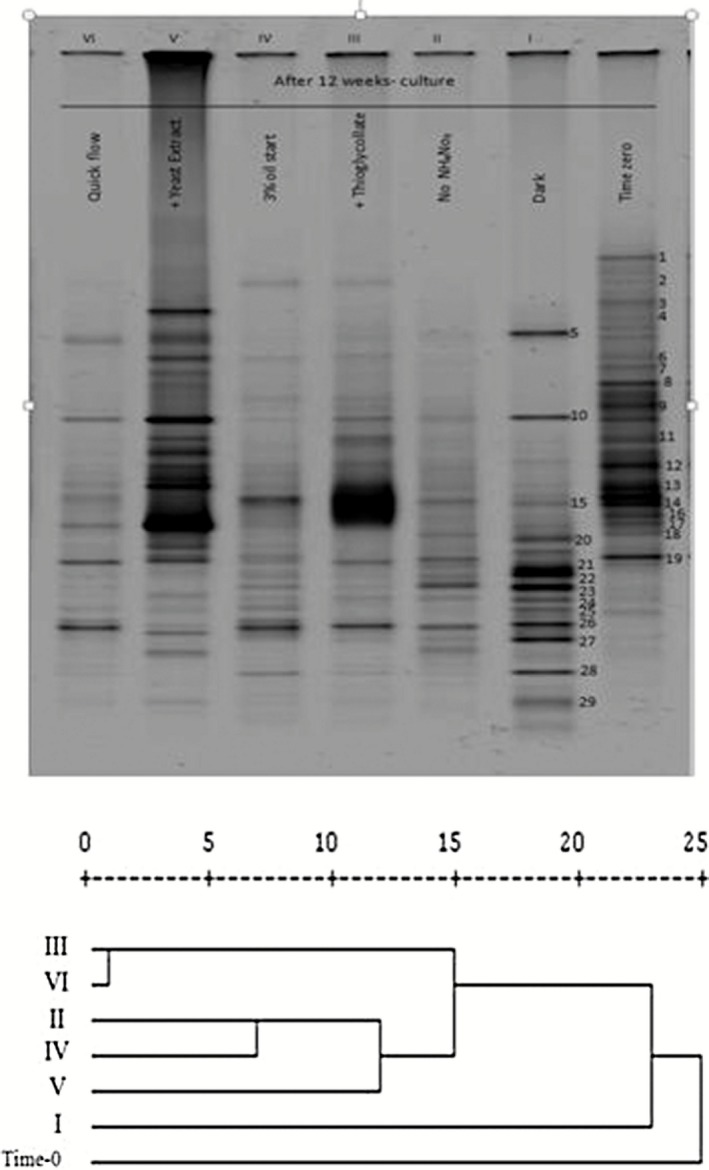
Upper: Typical DGGE profiles of 16S rRNA amplicons of total genomic DNA samples extracted from seawater in the reaction vessels of the six chemostats. For band identities see Table 5. Lower: Cluster analysis using Euclidean distances.

The typical denaturing gradient gel electrophoresis (DGGE) profiles in Fig. 6 show that 29 16S rDNA‐amplicon bands were recognized in the seawater samples of the six chemostat‐like units. However, sequencing was successful with 14 bands only. Sequences of the residual bands were of low quality, probably due to failure of clean multiple band separation. Table 5 shows that none of the hydrocarbonoclastic bacteria that had been isolated by the culture‐dependent method (Table 3) showed up in the list of taxa captured by the culture‐independent analysis. As will be discussed soon, such technical problems faced earlier investigators; their bases need to be studied. Even the bacterial classes in both lists were quite different. The culture‐independent approach captured among others, two phototrophic organisms, *Lyngbya aestuarii* and an uncultured *Chloroflexi bacterium*, which apparently had their origin in the microbial mat inoculum. Four *Alphaproteobacteria; Paracoccus pantotrophus, Maricaulis maris, Tistrella mobilis* and *Mesorhizobium thiogangeticum* as well as two *Flavobacteriales*; two *Gammaproteobacteria* and 1 each of the *Bacteoidates, Microgenomates*, *Tenericutes* and *Actinobacteria* were also detected. Several of those taxa had been recorded in the literature as hydrocarbonoclastic (see below).

**Table 5:** 16S rRNA gene sequencing of amplicon bands in Fig. 5

Band number	Total bases	Subdivision	Nearest GenBank match	Similarity %	Bases compared	Accession numbers
1	478	*Bacteroidetes*	Uncultured *Bacteroidetes* bacterium clone AH.KK	98	502/513	KP276699
4	229	*Verrucomicrobia*	Uncultured *Verrucomicrobia* bacterium clone Cy07‐41	96	250/260	KP276700
5	264	*Flavobacteriia*	Uncultured *Flavobacteriales* bacterium clone Clip 101	92	320/348	KP276701
6	509	*Flavobacteriia*	*Flavobacteriales* bacterium DG1510	97	523/537	KP276702
7	438	*Tenericutes*	*Tenericutes* bacterium P19x1ox‐fac	97	470/486	KP276703
10	513	*Gammaproteobacteria*	*Alcanivorax borkumensis* gene	100	513/513	KP276704
17	384	*Alphaproteobacteria*	*Paracoccus pantotrophus*	90	494/546	KP276705
19	335	*Cyanobacteria*	*Lyngbya aestuarii* CCY 961 clone CC8.	96	367/383	KP276706
20	454	*Alphaproteobacteria*	*Maricaulis maris* strain NBRC 102484	98	476/487	KP276707
21	379	*Cyanobacteria*	*Lyngbya aestuarii* PCC 7419	92	453/490	KP276708
22	443	*Alphaproteobacteria*	*Tistrella mobilis* strain SUVIK04	98	463/473	KP276709
25	391	*Alphaproteobacteria*	*Mesorhizobium thiogangeticum* strain XJB‐YJ18	94	454/485	KP276710
27	484	*Chloroflexi*	Uncultured *Chloroflexi* bacterium clone HAHS13.68	99	490/493	KP276711
**28**	439	Actinobacteria	Uncultured actinobacterium clone Paddy_16_4942	99	446/449	KP276712
**29**	369	*Gammaproteobacteria*	*Alkalispirillum mobile* strain DSM 12769	91	467/515	KP276713

Sequencing failed with bands 2,3,8,9,11,12,13,14,15,16,18,23, 24 and 26.

## Discussion

In the beginning, a few relevant facts need to be addressed. Organisms indigenous to a habitat should be able to colonize it easily. Furthermore, they should contribute significantly to activities in this habitat. Organisms not doing so should be regarded as allochthonous. A strain autochthonous in one environment may be allochthonous in another.

To recall, the objective of this contribution was to investigate the feasibility of bioaugmenting local oily desert soil and seawater samples with local microbial mats, for the purpose of soil and water bioremediation. Therefore, we monitored oil removal in bioaugmented batch and continuous cultures, and correlated it with the dynamics of the hydrocarbonoclastic bacterial populations in the studied cultures. Confirming earlier reports on bioaugmentation and biostimulation (Jimenez *et al*., 2007; Nikolopoulou and Kalogerakis, 2009), our results indicated that both practices were effective in bioremediating hydrocarbons, albeit to varying extents. The fact that individual autochthonous isolates consumed between one fifth and one third of oil implies that the total microbial consortia must be quite effective in cleaning suitable oily habitats. The results generally indicate that the microbial mats are more suitable bioaugmentation materials for bioremediation of hydrocarbon‐contaminated soil than seawater samples.

In batch cultures, bioaugmentation‐mediated oil removal was more effective in soil than in seawater samples. This is apparently due to the fact that soil contains more nutrients for microorganisms than seawater. Oil removal in seawater ceased 1 month after bioaugmentation, obviously due to the rather quick depletion of the limited nutrients and oxygen. The fact that the bacterial numbers decreased after an initial phase of increase coordinates with the typical growth curve in batch cultures. Judged by the culture stinky smell, anaerobiosis prevailed quite early in seawater, but not in soil cultures. In other words, bioremediation in seawater started under aerobic conditions, which turned anaerobic with time. In soil, on the other hand, aerobic conditions prevailed through the total incubation period. The results imply that batch culture bioremediation for seawater should not exceed 1 month. On the other hand, soil bioremediation should extend for several months. Interestingly, seawater batches, unlike soil batches, did not ‘welcome’ the autochthonous bacterial species of the mat inocula. Instead, the seawater batches were enriched with *M. hydrocarbonoclasticus*, a typical seawater autochthonous bacterium. In other words, the physicochemical parameters in soil, but not in seawater, were suitable for microorganisms indigenous to the mat habitat. This is surprising, since the mats *in situ* are frequently submerged in seawater during high tide. However, the natural resistance of such bacteria to being washed out by tidal movement coordinates with their failure to colonize seawater batches following mat bioaugmentation.

The facts that oil removal values in soil were higher than in seawater batches and that many of the typical mat bacteria appeared in the oily soil samples reflect and confirm the ready colonization of soil but not seawater by autochthonous mat bacteria. However, oil removal in soil seems to have been due to the collective activity of indigenous mat and indigenous soil bacteria. The dynamic changes of the microbial communities as described in this study confirm that, and probably coped with the types of intermediates of oil biodegradation at the time of analysis.

The short‐term continuous culture approach was adopted in this study for seawater remediation via mat bioaugmentation in an attempt to avoid the rather quick cell death in batch cultures. Meanwhile, it was proposed to couple bioaugmentation with biostimulation, as recommended by earlier workers (Odokuma and Dickson, 2003; El Fantroussi and Agathos, 2005). Although much more bacterial diversity in seawater was noted during continuous than batch culture, out of the seven identified hydrocarbonoclastic bacterial species, only *M. chlorophenolicum* seemed to have had its origin in the mat. Other species were indigenous seawater inhabitants. However, the culture‐independent analysis revealed a different list of bacteria, many of which belonged to the *Alphaproteobacteria*, *Gammaproteobacteria* and *Flavobacteriales*. Earlier researchers, too, found that *Gammaproteobacteria* (*Alcanivorax*, *Marinobacter*) and *Alphaproteobacteria* were ‘key players’ in oil degradation in contaminated Mexico beach sands (Kostka *et al*., 2011). The fact that culture‐dependent and culture‐independent approaches capture dissimilar bacterial taxa confirms and consolidates earlier reports from our (Al‐Awadhi *et al*., 2013) and other laboratories (Polz and Cavanaugh, 1998; Sipos *et al*., 2007).

The aerobic continuous culture approach was effective in oil removal under certain conditions; the following relevant recommendations may be made. First, to start the continuous fermentation with the highest tolerable oil concentration, i.e. not to feed it as smaller aliquots during the course of bioremediation. Second, to provide the system with vitamin‐containing natural products. In this context, vitamins have been reported earlier to enhance microbiological hydrocarbon biodegradation (Radwan and Al‐Muteirie, 2001; Al‐Mailem *et al*., 2013). Third, the water flow should be considered critically; too quick flow inhibits oil removal. No addition of nitrogenous compounds is needed, probably many of the inhabitants are diazotrophic, e.g. *Dietzia*, *Gordonia* and most of the hydrocabonoclastic bacteria (Dashti *et al*., 2015). Reducing substances such as thioglycollate do not seem to inhibit the bioremediation process, even though molecular oxygen is involved in the initial step of microbial attack on the hydrocarbon substrate (Ratledge, 1978; Radwan, 2009). The mats seem to harbour adequate oxygenic phototrophic inhabitants, which keep the cultures well aerated.

In conclusion, although local, environmental samples must be used for bioaugmentation, the physicochemical parameters in the targeted, contaminated site must be suitable for the inoculated microbial taxa. Should this not be the case, the bioaugmented bacteria would not contribute significantly to bioremediation. Specifically in this study, bioaugmentation of desert soil with costal mat is a typical autochthonous bioaugmentation practice. The mat bacteria showed up and exhibited dynamic behaviour in the soil. On the other hand, bioaugmentation of seawater with the same material merits the designation ‘allochthonous bioaugmentation’. With the exception of taxa naturally inhabiting both materials (see Table 1), mat bacteria failed to show up in seawater, which obviously was bioremediated via the typical seawater bacteria only. From a practical viewpoint, allochthonous bioaugmentation is obviously useless as a bioremediation approach.

## Experimental procedures

### Coastal microbial mats

Information on the microbial composition of such mats and their relation to the self‐cleaning of the Gulf is available in one of our earlier reports (Sorkhoh *et al*., 1992). The mats consist of phototrophic microorganisms, predominantly the filamentous cyanobacterium *Microcoleus* sp., which harbour in its filament sheaths millions of cells of hydrocarbonoclastic bacteria per gram fresh mat. Mat samples used in this study were freshly collected from the Sooq Sharq coast of Kuwait City (see Kuwait map in Fig. 1). The samples were transported in sterile conical flasks to the laboratory to be processed in the same day. Culture‐dependent counting (see below) revealed that each gram of fresh mat harboured 2.8 × 10^6^ hydrocarbonoclastic bacterial cells.

### Seawater and desert soil samples

Seawater samples from the Arabian Gulf and desert soil samples from stations at the north, middle and south of Kuwait (see the map in Fig. 1) were used for this bench scale bioremediation study. Seawater samples were collected from Subbyah, Kuwait Towers and Khiran areas, about 5 m offshore. Desert soil samples were collected from Kadma, Shuaybah and Wafra areas. The samples were transported to the laboratory and started to be processed in the same day. Three samples, 10 m apart, were collected from each site, pooled, mixed thoroughly and used in the bioremediation experiments, as described below. The water‐holding capacity of the soil samples ranged between 57.3 and 57.7%, w/w.

### Oil bioremediation in batch cultures

All experiments were done in triplicates. Fifty milliliter aliquots of pooled seawater were dispensed in 250 ml conical flasks. Pooled soil samples, 50 g, were also dispensed in 250 ml conical flasks and suspended in 50 ml aliquots of sterile tap water. Each flask received in addition 1%, w/v, weathered Kuwaiti light crude oil. To compare the behaviour of the inoculated microorganisms in the absence and presence of the inhabitant microorganisms, the flasks of one set were sterilized by autoclaving to kill the already existing microorganisms (designated sterile), and the flasks of another set were left unautoclaved (designated fresh). Each flask was inoculated with 1 ml (≡ 2 g mat) cell suspension prepared by homogenizing 200 g mat in 100 ml seawater. The flasks were sealed to avoid oil loss by volatilization and incubated at 30°C. At time zero and at monthly intervals, flasks were taken for residual oil recovery and quantitative determination, as well as for microbiological analysis. The mean values of the readings from the three replicates were determined and the standard deviations were calculated.

### Oil bioremediation in continuous cultures

This approach was used for bioremediating oily seawater samples only. For this, six identical chemostat‐like units were constructed, each consisting of a seawater vessel, leading successively to a culture vessel and a receiver vessel. The seawater vessel contained the freshly collected seawater to be continuously fed (by gravity effect) into the culture vessel. If not otherwise specified, the water flow rate was adjusted at 6 ml h^−1^. This rate was determined in preliminary experiments. Each culture vessel received at time zero 200 ml seawater which was inoculated only once with 1 ml mat suspension as bioaugmentation material. Five of the culture vessels received at time zero and every 2 weeks, 0.5 g aliquots (totally 3 g per vessel) of weathered Kuwaiti light crude oil. The sixth vessel received the whole 3 g oil in one lot at time zero. The six chemostat‐like units were set up to compare oil bioremediation as affected by six different biostimulation treatments:

I – To study the effect of light; the culture vessel of unit I was dark incubated by wrapping it in three successive layers of aluminum foil. The remaining five culture vessels were left exposed to day (about 13 h)–night (about 11 h) cycles.

II – To study the effect of added nitrogen fertilizers, culture vessel II was set up without added NH_4_NO_3_, unlike other culture vessels that contained 0.5%, w/v, NH_4_NO_3_.

III – To study the effect of the redox potential, one of the day–night exposed, NH_4_NO_3_‐containing culture vessels (unit III) was provided with 0.025%, w/v, thioglycollic acid. The remaining vessels did not receive this reducing substance.

IV – To study the feasibility of starting bioremediating using high‐oil concentration, instead of adding it in aliquots during incubation, one culture vessel (unit IV) received at time zero the whole 3 g crude oil in one lot, as described above.

V – To study the effect of vitamins, only one (unit V) of the day–night‐exposed, NH_4_NO_3_‐containing culture vessels received 0.2%, w/v, yeast extract.

VI – To study the effect of the seawater flow rate, the rate in unit VI was adjusted at 30 ml h^−1^; in the other five it was kept at 6 ml h^−1^. All the chemostat‐like units were incubated under room conditions for 12 weeks.

### Measurements of oil consumption

Residual hydrocarbons in the contents of individual chemostat‐like units were recovered at the end of the 12‐week incubation by extraction with three successive 20 ml portions of pentane. Extraction involved oil still adhering to the vessel walls and connecting tubes. The combined extract was raised to 60 ml using pure pentane, and 1 μl was analysed by gas liquid chromatography (GLC) using a Varian 3900 (USA) instrument equipped with an Flame Ionization Detector (FID), a Wall coated Open Tubular (WCOT)‐fused silica CP‐Sil 5 CB capillary column (Varian, USA), and a temperature program 45–310°C with temperature rising 10°C min^−1^, using N_2_ as a carrier gas. The detector temperature was 300°C and injector temperature 270°C. The percentage of oil consumption was calculated as the percentage reduction of total hydrocarbon peak areas in the GLC profiles based on the total areas of peaks in the GLC profiles at time zero. A similar method was used to determine crude oil consumption by individual bacterial isolates in batch cultures. A mineral medium (Sorkhoh *et al*., 1990, see below) which 1 g l^−1^ oil as a sole source of carbon and energy was used. Each flask was inoculated with 1 ml of bacterial suspension (one loopful in 5 ml water). Triplicates were prepared throughout. The flasks were incubated under room conditions for 14 days.

### Culture‐dependant analysis of hydrocarbonoclastic bacteria

The conventional dilution plating method was used for counting hydrocarbonoclastic bacteria in the cultures. A solid mineral medium (Sorkhoh *et al*., 1990) with oil vapour as the sole source of carbon and energy was used. The medium consisted of (g l^−1^): 30.0 NaCl 5.0 NaNO_3_, 0.56 KH_2_PO_4_, 0.86 Na_2_HPO_4_, 0.17 K_2_SO_4_, 0.37 MgSO_4_.7H_2_O, 0.7 CaCl_2_.2H_2_O, 2.5 ml of trace element mixture (g l^−1^): 2.3 ZnSO_4_, 1.8 MnSO_4_, 0.6 H_3_BO_3_, 1.0 CuSO_4_, 0.4 Na_2_MoO_4_, 0.4 CoCl_2_, 0.7 KI, 1.0 EDTA, 0.4 FeSO_4_, 0.004 NiCl_2_, pH 7.0. Agar, 20 g l^−1^ was added for medium solidification. Each plate lid was provided with a filter paper impregnated with 2 ml crude oil, and the covered plates were sealed after inoculation with 0.25 ml of each inoculum. The volatile oil vapour was the sole source of carbon and energy available to the developing colonies. The plates were incubated at 30°C for 10 days. The total colony‐forming units (cfu) were counted, and the mean values ± standard deviation values calculated per millilitre seawater or gram soil. Parallel plates were pooled and colonies with identical morphologies were counted and their percentage of the total calculated. For characterization of individual bacterial isolates, their 16S rRNA gene sequences were compared with the closest sequences in the GenBank database. The PrepMan Ultra Kit (Applied Biosystems, Foster City, CA, USA) was used to extract genomic DNA from pure isolates, and the 16S rRNA genes were amplified by polymerase chain reaction (PCR) using the GM5F (5′‐CCTACGGGAGGCAGCAG‐3′) and 907R (5′‐ CCCCGTCAATTCMTTTGAGTTT‐3′) primers (Santegoeds *et al*., 1998). The PCR products were purified using the QIA quick PCR purification kit (Qiagen, Valencia, CA, USA) in order to remove the Taq polymerase, primers and deoxy nucleotide tri phosphates (dNTPs). Partial sequencing of the 16S rRNA genes was performed using the BigDye version Terminator Kit (Applied Biosystems, Warrington, UK). Pure template DNA samples were processed in the 3130 × l genetic analyser (Applied Biosystems, Foster City, CA, USA). Sequencing analysis version 5.2 software (Applied Biosystems, Foster City, CA, USA) was used to analyse the results. Sequences were subjected to basic local alignment search tool analysis with the National Center for Biotechnology Information (Bethesda, MD, USA) GenBank database (Altschul *et al*., 1997). A phylogenetic tree was constructed by neighbour‐joining including bootstrap analysis using paup* v.4 (Swafford). Bootstrap proportions were used on 2000 replicates.

### Culture‐independent analysis of total bacteria

To analyse the total bacterioflora in various cultures, the total genomic DNA was extracted using the Rapid Water DNA Isolation Kit [MO‐BIO, Carlsbad, CA (for media) and the Fast DNA Spin for Soil Kit (MP Biomedicals, LIC., France]. The 16S rRNA genes in the genomic DNA samples were partially amplified using the universal primer pair GM5F (with a G (guanine) C (cytosine) clamp) and 907R (Schäfer and Muyzer, 2001). The resulting amplicons were resolved by parallel DGGE using the DCode Universal Mutation Detection System (Bio‐Rad, California, USA). The denaturant concentrations were 45–60%. Electrophoresis was run under constant voltage of 50 V at 60°C for 16 h. Gels were stained with SYBR Green (Invitrogen, USA) in 1xTAE buffer (1:100,000) for 30 min and inspected using a Dark Reader transilluminator (Clare Chemical Research, CO, USA). The bands were transformed into binary matrix; the presence of bands was given the weight of ‘1’ and their absence ‘0’. The binary matrix produced was analysed using cluster analysis, and a dendogram was plotted. Gel bands carrying 16S ribosomal DNA (rRNA) fractions were excised and stored overnight in 50 μl molecular water (Sigma, UK) at 4°C to elute the DNA. One microliter of the eluted DNA was amplified using the above primer pair, sequenced, and the sequences were compared with those in the GenBank database.

## Conflict of interest

The authors declare that they have no conflict of interests.

## References

[mbt212326-bib-0001] Al‐Awadhi, H. , Al‐Daher, R. , ElNavavy, A. , and Balba, M.T. (1996) Bioremediation of oil‐contaminated soil in Kuwait: landfarming to remediate oil‐contaminated soil. J Soil Contam 5: 243–260.

[mbt212326-bib-0002] Al‐Awadhi, H. , Dashti, N. , Khanafer, M. , Al‐Mailem, D. , Ali, N. , and Radwan, S.S. (2013) Bias problems in culture‐independent analysis of environmental bacterial communities: a representative study on hydrocarbonoclastic bacteria. Springer Plus 2: 369–379. 24040582 10.1186/2193-1801-2-369 PMC3769543

[mbt212326-bib-0003] Al‐Mailem, D.M. , Eliyas, M. , and Radwan, S.S. (2013) Enhanced bioremediation of oily hypersaline coastal areas in Kuwait via vitamin‐fertilization. Environ Sci Pollut Res 21: 3386–3394. 10.1007/s11356-013-2293-6 24243095

[mbt212326-bib-0004] Altschul, S.F. , Madden, T.L. , Schäffer, A.A. , Zhang, J. , Zheng, Z. , Miller, W. , and Lipman, D.J. (1997) Gapped BLAST and PSI‐BLAST: a new generation of protein database search programs. Nucleic Acid Res 25: 3389–3402. 9254694 10.1093/nar/25.17.3389 PMC146917

[mbt212326-bib-0005] Atlas, R.M. (1991) Microbial hydrocarbon degradation‐bioremediation of oil spills. J Chem Technol Biotechnol 52: 149–156.

[mbt212326-bib-0006] Atlas, R.M. , and Bartha, R. (1998) Microbial Ecology: Fundamentals and Applications, 4th edn Canada: Benjamin/Cummings Publishing.

[mbt212326-bib-0007] Atlas, R.M. , and Pramer, D. (1990) Focus on bioremediation. ASM News 56: 352–353.

[mbt212326-bib-0008] Bento, F.M. , Camargo, F.A.O. , Okeke, B.C. , and Frankenberger, W.T. (2005) Comparative bioremediation of soils contaminated with diesel oil by natural attenuation, biostimulation and bioaugmentation. Biores Technol 96: 1049–1055. 10.1016/j.biortech.2004.09.008 15668201

[mbt212326-bib-0009] Bossert, I. , and Bartha, R. (1984) The fate of petroleum in soil ecosystems In Petroleum Microbiology. Atlas R.M. (ed.). New York: Macmillan Publishing, pp. 434–476.

[mbt212326-bib-0010] Coppotelli, B.M. , Ibarrolaza, A. , Del Panno, M.T. , and Morelli, I.S. (2008) Effects of the inoculants strain *Sphingomonas paucimobilis* 20006 FA on soil bacterial community biodegradation in phenanthrene – contaminated soil. Microb Ecol 55: 173–183. 17694405 10.1007/s00248-007-9265-7

[mbt212326-bib-0011] Dashti, N. , Eliyas, M. , Khanafer, M. , Sorkhoh, N.A. , and Radwan, S.S. (2015) Most hydrocarbonoclastic bacteria in the total environment are diazotrophic which highlights their value in hydrocarbon‐bioremediation. Microb Environ 30: 70–75. 10.1264/jsme2.ME14090 PMC4356466 25740314

[mbt212326-bib-0012] DiGregorio, S. , Castglione, M.R. , Gentini, A. , and Lorenzi, R. (2015) Biostimulation of the autochthonous bacterial community and bioaugmentation of selected bacterial strains for the depletion of polycyclic aromatic hydrocarbons in a historically contaminated soil. Geophys Res Astracts 17: id: 14690.

[mbt212326-bib-0013] Domde, P. , Kapley, A. , and Purohit, H.J. (2007) Impact of bioaugmentation with a consortium of bacteria on the remediation of wastewater‐containing hydrocarbons. Enviro Sci Pollut Res 14: 7–11. 10.1065/espr2006.11.358 17352122

[mbt212326-bib-0014] El Fantroussi, S. , and Agathos, S.N. (2005) Is bioaugmentation a feasible strategy for pollutant removal and site remediation? Current Opin Microbiol 8: 268–275. 10.1016/j.mib.2005.04.011 15939349

[mbt212326-bib-0015] Goldstein, J.F. , Mallory, L.M. , and Alexander, M. (1985) Reason for possible failure of inoculation to enhance biodegradation. Appl Environ Microbiol 50: 917–983. 10.1128/aem.50.4.977-983.1985 PMC291779 4083891

[mbt212326-bib-0016] Head, I.M. (1998) Bioremediation: a response to gross environmental abuse. Trend Biotechnol 11: 599–608.

[mbt212326-bib-0017] Hosakawa, R. , Nagai, M. , Morikawa, M. , and Okayama, H. (2009) Autochthonous bioaugmentation and its possible application to oil spills. World J Microbiol Biotechnol 25: 1519–1528.

[mbt212326-bib-0018] Jimenez, N. , Vinas, M. , Bayona, J.M. , Albaiges, J. , and Solanas, A.M. (2007) *The Prestige* oil spill: bacterial community dynamics during a field biostimulation assay. Appl Microbiol Biotechnol 77: 935–945. 17943279 10.1007/s00253-007-1229-9

[mbt212326-bib-0019] Kapley, A. , and Purohit, H.J. (2001) Tracking of phenol degrading genotype. Environ Sci Pollut Res 8: 89–90. 10.1007/BF02987299 11400643

[mbt212326-bib-0020] Korda, A. , Santas, P. , Tenente, A. , and Santas, R. (1997) Petroleum hydrocarbon bioremediation: sampling and analytical techniques, in situ treatments and commercial microorganisms currently used. Appl Microbiol Biotechnol 48: 677–686. 9457796 10.1007/s002530051115

[mbt212326-bib-0021] Kostka, J.E. , Prakash, O. , Overholt, W.A. , Green, S.J. , Freyer, G. , Canion, A. , *et al* (2011) Hydrocarbon‐degrading bacteria and the bacterial community response in gulf of Mexico beach sands impacted by the deepwater horizon oil spill. Appl Environ Microbiol 77: 7962–7974. 21948834 10.1128/AEM.05402-11 PMC3208977

[mbt212326-bib-0022] Kuiper, I. , Lagendijk, E.L. , Bloemberg, G.V. , and Lugtenberg, J.J. (2004) Rhizoremediation: a beneficial plant‐microbe interaction. Molec Plant‐Microb Interac 17: 6–15. 10.1094/MPMI.2004.17.1.6 14714863

[mbt212326-bib-0023] Leahy, J.G. , and Colwell, R.R. (1990) Microbial degradation of hydrocarbons in the environment. Microbiol Rev 54: 305–315. 2215423 10.1128/mr.54.3.305-315.1990 PMC372779

[mbt212326-bib-0024] Margesin, R. , and Schinner, F. (1998) Oil biodegradation potential in alpine habitats. Arctic Alp Res 30: 262–265.

[mbt212326-bib-0025] Moharikar, A. , Kapley, A. , and Purohit, H.J. (2003) Detection of dioxygenase genes present in various activated sludge. Environ Sci Pollut Res 10: 373–376. 10.1065/espr2003.07.164 14690027

[mbt212326-bib-0026] Namkoong, W. , Hwang, E.Y. , Park, J.S. , and Choi, J.Y. (2002) Bioremediation of diesel‐contaminated soil with composting. Environ Pollut 119: 23–31. 12125726 10.1016/s0269-7491(01)00328-1

[mbt212326-bib-0027] Nikolopoulou, M. , and Kalogerakis, N. (2009) Biostimulation strategies for fresh and chronically polluted marine environments with petroleum hydrocarbons. J Chem Technol Biotechnol 84: 802–807.

[mbt212326-bib-0028] Nikolopoulou, M. , Eickenbusch, P. , Pasadakis, N. , Venieri, D. , and Kalogerakis, N. (2013a) Microcosm evaluation of autochthonous bioaugmentation to combat marine oil spills. New Biotechnol 30: 734–742. 10.1016/j.nbt.2013.06.005 23835403

[mbt212326-bib-0029] Nikolopoulou, M. , Pasadakis, N. , and Kalogerakis, N. (2013b) Evaluation of autochthonous bioaugmentation and biostimulation during microcosm‐simulated oil spills. Mar Pollut Bull 72: 165–173. 23660443 10.1016/j.marpolbul.2013.04.007

[mbt212326-bib-0030] Odokuma, L.O. , and Dickson, A.A. (2003) Bioremediation of a crude oil polluted tropical rain forest soil. Glob J Environ Sci 2: 29–40.

[mbt212326-bib-0031] Piskonen, R. , and Itävaara, M. (2004) Evaluation of chemical pretreatment of contaminated soil for improved PAH bioremediation. Appl Microbiol Biotechnol 65: 627–634. 15293029 10.1007/s00253-004-1679-2

[mbt212326-bib-0032] Polz, M.F. , and Cavanaugh, C.M. (1998) Bias in template‐to‐product ratios in multitemplate PCR. Appl Environ Microbiol 64: 3724–3730. 9758791 10.1128/aem.64.10.3724-3730.1998 PMC106531

[mbt212326-bib-0033] Radwan, S. , and Sorkhoh, N. (1993) Lipids of n‐alkane‐utilizing microorganisms and their application potential. Adv Appl Microbiol 39: 29–90.

[mbt212326-bib-0034] Radwan, S.S. (1991) The Gulf oil spill. Nature 350: 456.2014048

[mbt212326-bib-0035] Radwan, S.S. (2009) Phytoremediation for oily desert soils In Advances in Applied Bioremediation. Singh A., Kuhad R.C., and Ward O.P. (eds). Berlin: Springer, pp. 279–298.

[mbt212326-bib-0036] Radwan, S.S. , and Al‐Muteirie, A.S. (2001) Vitamin requirements of hydrocarbon‐utilizing soil bacteria. Microbiol Res 155: 301–307. 11297361 10.1016/S0944-5013(01)80008-2

[mbt212326-bib-0037] Radwan, S.S. , Sorkhoh, N.A. , El‐Nemr, I. , and El‐Desouky, A.F. (1997) A feasibility study on seeding as a bioremediation practice for the oily Kuwaiti desert. J Appl Microbiol 83: 353–358.

[mbt212326-bib-0038] Rahman, K.S. , Thahira‐Rahman, J. , Lakshmanaperumalsamy, P. , and Banat, I.M. (2002) Towards efficient crude oil degradation by a mixed bacterial consortium. Biores Technol 85: 257–261. 10.1016/s0960-8524(02)00119-0 12365493

[mbt212326-bib-0039] Ratledge, C. (1978) Degradation of aliphatic hydrocarbons In Developments in Biodegradation of Hydrocarbons, Vol. 1 Watkinson I. (ed.). Essex: Applied Science, pp. 1–45.

[mbt212326-bib-0040] Rosenberg, E. (1993) Exploiting microbial growth on hydrocarbons – new markets. Trend Biotechnol 11: 419–424.

[mbt212326-bib-0041] Rosenberg, E. (2006) Hydrocarbon‐oxidizing bacteria In The Prokaryotes, a Handbook on the Biology of Bacteria, Vol. 2, 3rd edn Dworkin M., Falkow S., Rosenberg E., Schleifer K.H., and Stackebrandt E. (eds). Berlin: Springer, pp. 564–577.

[mbt212326-bib-0042] Santegoeds, C.M. , Ferdelman, T.G. , Muyzer, G. , and Beer, D. (1998) Structural and functional dynamics of sulfate‐reduction populations in bacterial biofilms. Appl Environ Microbiol 64: 3731–3739. 9758792 10.1128/aem.64.10.3731-3739.1998 PMC106533

[mbt212326-bib-0043] Schäfer, H. , and Muyzer, G. (2001) Denaturing gradient gel electrophorasis in marine microbial ecology. Method Microbiol 30: 425–468.

[mbt212326-bib-0044] Seklemova, E. , Pavlova, A. , and Kovacheva, K. (2001) Biostimulation‐based bioremediation of diesel fuel: field demonstration. Biodeg 12: 311–316. 11995824 10.1023/a:1014356223118

[mbt212326-bib-0045] Sipos, R. , Szekely, A.J. , Palatinsky, M. , Revesz, S. , Marialigeti, K. , and Nicolausz, M. (2007) Effect of primer mismatch, annealing temperature and PCR cycle number on 16S rRNA gene‐targeting bacterial community analysis. FEMS Microbiol Ecol 60: 341–350. 17343679 10.1111/j.1574-6941.2007.00283.x

[mbt212326-bib-0046] Sorkhoh, N. , Al‐Hasan, R. , Radwan, S. , and Höpner, T. (1992) Self‐cleaning of the Gulf. Nature 350: 109.

[mbt212326-bib-0047] Sorkhoh, N.A. , Ghannoum, M.A. , Ibrahim, A.S. , Stretton, R.J. , and Radwan, S.S. (1990) Crude oil and hydrocarbon degrading strains of *Rhodococcus rhodochrous* isolated from soil and marine environments in Kuwait. Environ Pollut 65: 1–17. 15092275 10.1016/0269-7491(90)90162-6

[mbt212326-bib-0048] Ueno, A. , Ito, Y. , Yumoto, I. , and Okuyama, H. (2007) Isolation and characterization of bacteria from soil contaminated with diesel oil and the possible use of these in autochthonous bioaugmentation. World J Microbiol Biotechnol 23: 1739–1745. 10.1007/s11274-007-9423-6 27517830

[mbt212326-bib-0049] Van Limbergen, H. , Top, E.M. , and Verstraete, W. (1998) Bioaugmentation in activated sludge: current features and future perspectives. Appl Microbiol Biotechnol 50: 16–23.

[mbt212326-bib-0050] Vecchioli, G.I. , Del Panno, M.T. , and Painceira, M.T. (1990) Use of selected autochthonous soil bacteria to enhance degradation of hydrocarbons in soil. Environ Pollut 67: 249–258. 15092212 10.1016/0269-7491(90)90190-n

[mbt212326-bib-0051] Weber, W.J., Jr , and Corseuil, H.K. (1994) Inoculation of contaminated subsurface soils with enriched indigenous microbes to enhance bioremediation rates. Water Res 28: 1407–1414.

